# Supplementary material for: The causal role of circulating inflammatory markers in osteoporosis: a bidirectional Mendelian randomized study
Source: Front Immunol. 2024 Jul 18;15:1412298. doi: 10.3389/fimmu.2024.1412298 (PMC11291241; doi:10.3389/fimmu.2024.1412298)
Supplement: Supplementary file 1 [file DataSheet_1.zip › supplementary material/OSTEOPOROSIS-CIM/GCST90274809.tsv.gz_forest.pdf]

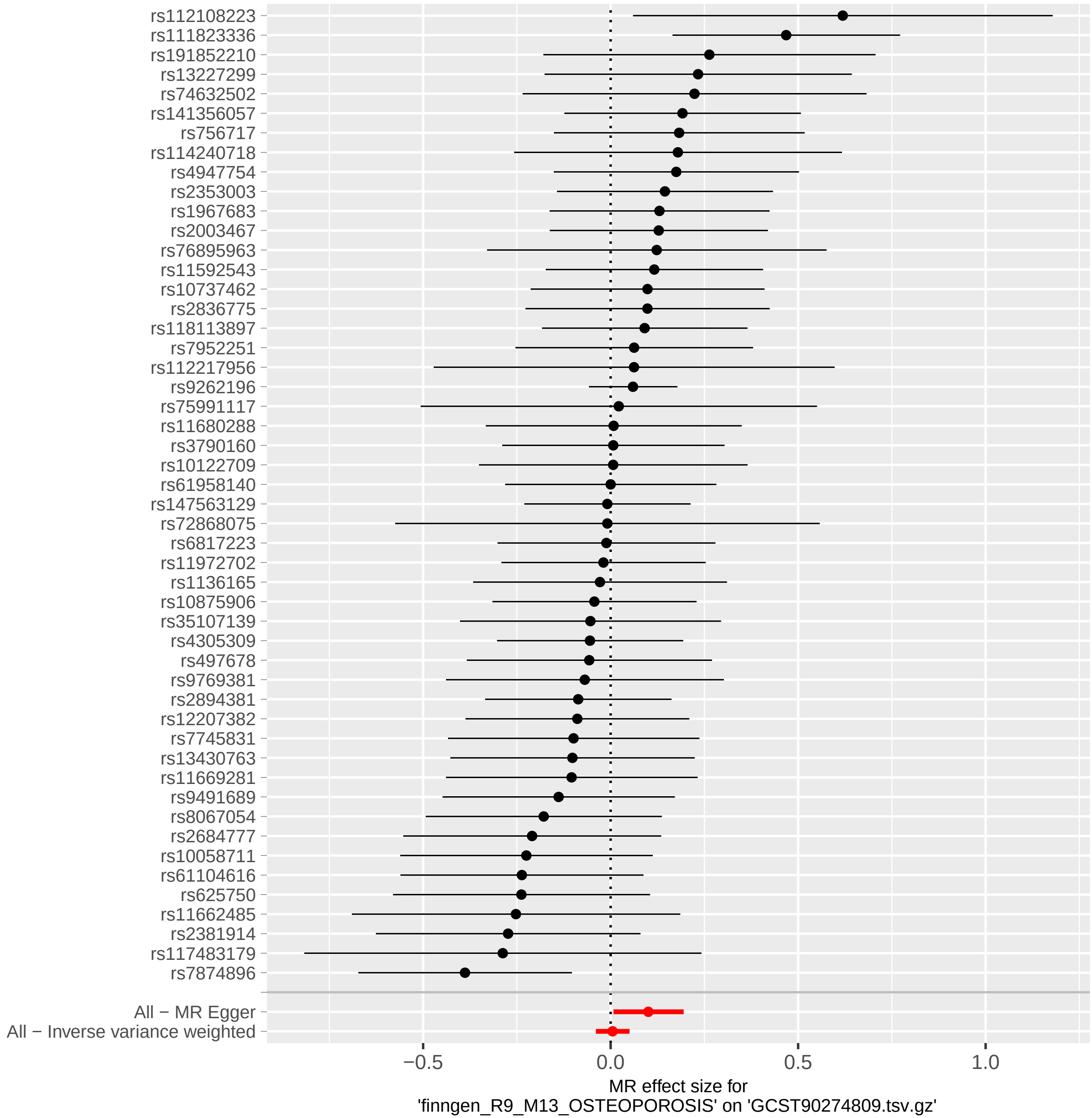
rs112108223
rs111823336
rs191852210
rs13227299
rs74632502
rs141356057
rs756717
rs114240718
rs4947754
rs2353003
rs1967683
rs2003467
rs76895963
rs11592543
rs10737462
rs2836775
rs118113897
rs7952251
rs112217956
rs9262196
rs75991117
rs11680288
rs3790160
rs10122709
rs61958140
rs147563129
rs72868075
rs6817223
rs11972702
rs1136165
rs10875906
rs35107139
rs4305309
rs497678
rs9769381
rs2894381
rs12207382
rs7745831
rs13430763
rs11669281
rs9491689
rs8067054
rs2684777
rs10058711
rs61104616
rs625750
rs11662485
rs2381914
rs117483179
rs7874896
All − MR Egger
All − Inverse variance weighted
−0.5
0.0
0.5
1.0
MR effect size for
'finngen_R9_M13_OSTEOPOROSIS' on 'GCST90274809.tsv.gz'
